# Age over sex: evaluating gut microbiota differences in healthy Chinese populations

**DOI:** 10.3389/fmicb.2024.1412991

**Published:** 2024-06-21

**Authors:** Jiacheng Wu, Hexiao Shen, Yongling Lv, Jing He, Xiaotian Xie, Zhiyue Xu, Pengcheng Yang, Wei Qian, Tao Bai, Xiaohua Hou

**Affiliations:** ^1^Division of Gastroenterology, Union Hospital, Tongji Medical College, Huazhong University of Science and Technology, Wuhan, Hubei, China; ^2^School of Life Science, Hubei University, Wuhan, Hubei, China

**Keywords:** gut microbiota, enterotype, age, sex, healthy populations

## Abstract

Age and gender have been recognized as two pivotal covariates affecting the composition of the gut microbiota. However, their mediated variations in microbiota seem to be inconsistent across different countries and races. In this study, 613 individuals, whom we referred to as the “healthy” population, were selected from 1,018 volunteers through rigorous selection using 16S rRNA sequencing. Three enterotypes were identified, namely, *Escherichia–Shigella*, mixture (*Bacteroides* and *Faecalibacterium*), and *Prevotella*. Moreover, 11 covariates that explain the differences in microbiota were determined, with age being the predominant factor. Furthermore, age-related differences in alpha diversity, beta diversity, and core genera were observed in our cohort. Remarkably, after adjusting for 10 covariates other than age, abundant genera that differed between age groups were demonstrated. In contrast, minimal differences in alpha diversity, beta diversity, and differentially abundant genera were observed between male and female individuals. Furthermore, we also demonstrated the age trajectories of several well-known beneficial genera, lipopolysaccharide (LPS)-producing genera, and short-chain fatty acids (SCFAs)-producing genera. Overall, our study further elucidated the effects mediated by age and gender on microbiota differences, which are of significant importance for a comprehensive understanding of the gut microbiome spectrum in healthy individuals.

## Introduction

The gut microbiota has coevolved with the human body, and its homeostasis plays a pivotal role in health, including education of the host immune system, protection against pathogens, and regulation of intestinal endocrine functions (Hou et al., 2022). The imbalances in the gut microbiota have been linked to a range of diseases, such as inflammatory bowel disease, allergies, and autoimmune diseases (Duvallet et al., 2017). Moreover, disruptions in the gut microbiota are also related to age-related diseases, such as Alzheimer’s disease, vascular dementia, and other neurodegenerative disorders (Chandra et al., 2023). Extensive studies targeting disease-related microbiota have implicated increasing microbial components in various pathologies, recognizing them as potential biomarkers. For instance, fecal microbial markers have been widely studied for colorectal cancer screening (Wong and Yu, 2019). However, the translation of microbiota research into clinical practice is still limited due to multiple challenges, especially the difficulty in precisely defining “healthy” microbiota, including the well-known probiotics and opportunistic pathogens. Therefore, elucidating the gut microbiota spectrum within healthy individuals is of great significance for targeting the microbiota to predict and treat associated diseases.

Age and sex have emerged as pivotal covariates in depicting the gut microbiome (Zhernakova et al., 2016; Zhang et al., 2021), with several reports highlighting their potential role in microbiota differences (de la Cuesta-Zuluaga et al., 2019; Zhang et al., 2021). However, inconsistent differences have been widely reported in various studies of microbiota related to age and gender. For example, no significant differences were observed in the alpha diversity of the gut microbiota between men and women or among age groups in a healthy Japanese cohort with 277 subjects aged 20–89 years (Takagi et al., 2019). Conversely, in another cohort of 1,741 Chinese adults, sex accounted for the majority of microbial variance (Zhang et al., 2021). Intriguingly, in a previous study encompassing adult cohorts from four different nations, significant correlations between microbial diversity and age and sex were observed in American, British, and Colombian populations, while no significant associations between alpha diversity and age and sex were observed in Chinese individuals (de la Cuesta-Zuluaga et al., 2019).

Given the significance of delineating the gut microbiota spectrum in healthy individuals and the uniqueness of age- and sex-dependent microbiota across different countries and races, the present study recruited 1,018 Chinese volunteers. Through rigorous exclusion criteria, 613 individuals, whom we refer to as the “healthy” population, were used for subsequent analysis. Overall, our results provide insights into the intricate interplay between age and sex and the gut microbiome and elucidate their effect size on microbiota differences, thereby providing support for a comprehensive depiction of the gut microbiota spectrum in healthy individuals.

## Materials and methods

### Study description and population

To investigate the age- and sex-related trajectories of the gut microbiota in healthy Chinese individuals, we recruited 1,018 volunteers from China. The metadata (Supplementary Table S1) were collected through a questionnaire, including demographic information (age, sex, blood type, and BMI), lifestyle (smoking, alcohol drinking, exercise frequency, sleep duration, and sleep procrastination), dietary information (dietary regularity and dietary preference), mental stress frequency, and recent gastrointestinal manifestations (stool type, foul defecations, stink farts, and ozostomia). A total of 675 individuals lacking evidence of factors that could affect gut microbiota were identified based on a lengthy list of exclusion criteria; we will refer to them here as “healthy” (Supplementary Table S2). Exclusion criteria included individuals who used antibiotics or probiotics prior to 3 months of study participation, individuals with a history of fecal microbiota transplantation, individuals who are pregnant or lactating, individuals with a history of psychiatric disorder (e.g., anxiety and insomnia), individuals with a history of gastrointestinal symptoms or disorders (e.g., inflammatory bowel disease and irritable bowel syndrome), and individuals with a history of breast or genital system problems. Participants with other factors that could affect intestinal motility or gut microbiota, as evaluated by researchers, were also excluded. After further filtering of the samples lacking age and sex information, 613 healthy samples were used for subsequent analysis (Supplementary Table S3). Correlation between the metadata was measured by Spearman’s rank correlation with the psych R package v2.0.7 (Supplementary Tables S12, S13). A *p*-value of <0.05 was considered significant.

### Sample collection and 16S rRNA sequencing

Fecal samples were collected following a standardized procedure: participants were given detailed instructions. Following the instructions, participants collected the samples by themselves and stored the samples in home freezers or iceboxes; samples were transported to the research laboratory using a cold chain within 24 h; samples were then well homogenized, aliquoted, and stored at −80°C until further analyses. Microbial DNA was extracted from feces using the MP FastDNA Spin Kit for Feces (MP Biomedicals, Santa Ana, CA, United States) following the manufacturer’s instructions. The V3–V4 variable regions of the 16 S rRNA gene were amplified by PCR with the primers 314\F: CCTAYGGGRBGCASCAG and 806 R: GGACTACNNGGGTATCTAAT. The PCR product was evaluated using a 1.5% gel electrophoresis and purified by magnetic beads (Yeasen, Shanghai, China). The purified amplicons were sequenced using paired-end sequencing (PE 250) on an Illumina NovaSeq6000 platform. The raw sequencing reads for all raw datasets were subjected to reference-based chimera filtering using VSEARCH v2.10.3 (Rognes et al., 2016). Chimeric-filtered sequences were assigned to operational taxonomic units (OTUs) by OTU picking using the QIIME pipeline (Caporaso et al., 2010). Sequences were clustered using UCLUST (Edgar, 2010) into OTUs (≥ 97% similarity) based on the SILVA 132 database (Quast et al., 2013; Supplementary Table S4).

### Diversity analysis

To estimate the alpha diversity of the microbiota, observed species, Shannon, Simpson, Pielou, Ace, and Chao1 indices were calculated using the vegan R package v3.6.2 (Oksanen et al., 2024) (Supplementary Table S15). Furthermore, β diversity was estimated by using the Bray–Curtis distance and was calculated using the vegan R package and represented through principal coordinate analysis (PCoA).

### Enterotype analysis

The enterotype clustering was performed as previously described (Arumugam et al., 2011; Lu et al., 2021). Briefly, according to the relative abundance of each genus in each sample, Jensen–Shannon divergence (JSD) was calculated by using the “dist.JSD” function coded in R.^1^ Based on the obtained distance matrix, the 613 samples were clustered using partitioning around medoids (PAMs) clustering by using the “pam” function in the cluster R package v1.14.2 (Brock et al., 2008). The optimal number of clusters was chosen by maximizing the Calinski–Harabasz (CH) index, which is calculated by the “index.G1” function in the clusterSim R package v0.15–3 (Dudek and Walesiak, 2020; Supplementary Table S16). The result of clustering was visualized on the PCoA plot by the ade4 R package v1.7–15 (Brock et al., 2008). To identify the driving genera of each enterotype, random forest analysis with 10-time 5-fold cross-validation was performed (Lu et al., 2021) using the randomForest R package v4.6–15 (Breiman et al., 2022; Supplementary Table S17).

### Permutational multivariate analysis of variance (PERMANOVA)

To identify the covariates explaining the microbial difference, PERMANOVA was performed at the OTU, species, and genus levels based on the Bray–Curtis distance (Supplementary Table S14). The pseudo-F statistics and *p*-values were calculated using the “adonis” function from the vegan R package based on 9,999 permutations. The cutoff was set at a *p*-value of <0.05.

### Multivariate association with linear models (MaAsLin)

To evaluate whether there were robust age or sex differences in microbial composition when adjusting for the effects of other covariates, MaAsLin2 R package v1.8.0^2^ was used to treat each variable as a fixed effect (Supplementary Tables S18–20). The above-identified variables in PERMANOVA analysis were included in the models. The Benjamini–Hochberg method was used to adjust for multiple testing, and an FDR of <0.1 was considered significant.

### Statistical analyses

The continuous variables (that is, alpha diversity, beta diversity, and relative abundance) between the two groups were compared using a two-sided Wilcoxon rank-sum test, while the continuous variables among multiple groups were compared using the Kruskal–Wallis test. Data were presented as the mean ± standard error of the mean (SEM). A *p*-value of <0.05 was considered significant. The dynamic change curves of specific microbial genera with age and sex are fitted using locally weighted regression (loess) with the ggplot2 R package v3.4.4.

## Results

### An overview of the cohort and analysis of enterotypes

To delineate the gut microbiome landscape in a healthy Chinese population, 1,018 participants with an overall equivalent ratio were recruited from our cohort (Supplementary Table S1). After filtering the samples that we referred to as “healthy” according to the selection criteria (see Materials and methods), 613 healthy individuals (male: 358 and female: 255) were identified for further analyses (Supplementary Table S3). This cohort mainly consists of Han individuals (Han: 98.37%) from 18 provinces in China, with ages ranging from 1 to 99 years old.

**Figure 1:**
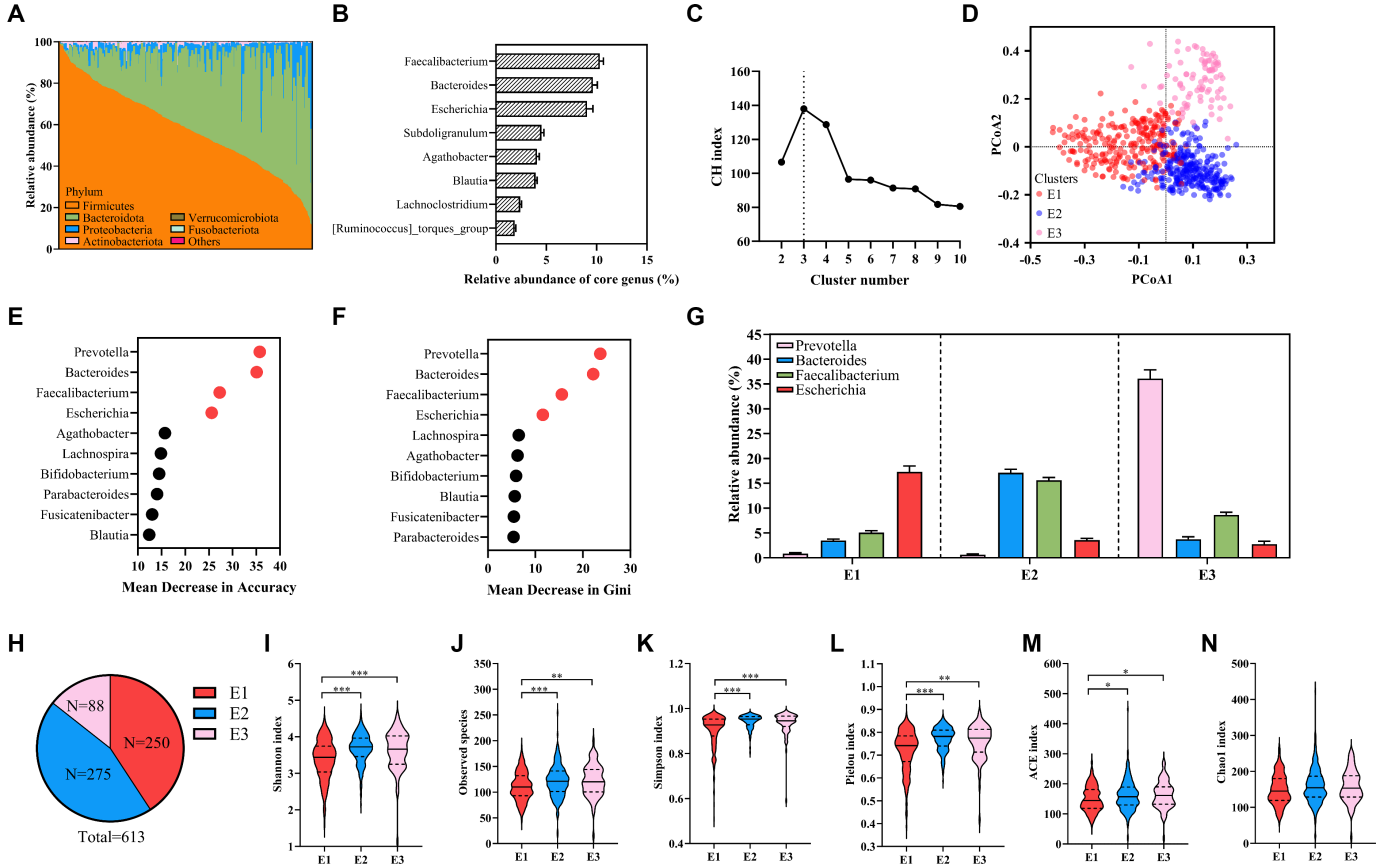
An overview of the cohort and analysis of enterotypes. **(A)** The relative abundance of the top six bacteria in 613 healthy samples at the phylum level. **(B)** The relative abundance of the eight core genera. **(C)** The CH Index. **(D)** PCoA plot showing three enterotypes. **(E,F)** The mean decrease in accuracy **(E)** and the mean decrease in Gini **(F)** of genera from random forest models classifying enterotypes. **(G)** The relative abundances of representative genera of enterotypes. **(H)** The number of each enterotype in 613 healthy samples. **(I–N)** Alpha diversity is evaluated by the Shannon Index **(I)**, observed species **(J)**, the Simpson Index **(K)**, the Pielou Index **(L)**, the ACE Index **(M)**, and the Chao1 Index **(N)**. Kruskal–Wallis test. Values are mean ± SEM. ^*^p < 0.05, ^**^p < 0.01, ^***^p < 0.001.

Consistent with the previous studies (De Filippo et al., 2010; Qin et al., 2010), the phyla Firmicutes, Bacteroidota, Proteobacteria, and Actinobacteria were found to be predominant in all samples (Figure 1A). A total of eight genera were observed in >90% of samples, with average relative abundances >0.1% (the core microbiota, Figure 1B). With the exception of *Subdoligranulum*, these genera matched the core gut microbiota of healthy Chinese individuals who resided in Guangdong province in 2008 (He et al., 2018). Four of these core genera overlapped with the top nine most abundant fecal genera in another Chinese cohort, which included 314 healthy individuals from nine provinces (Zhang et al., 2015). Additionally, four of the core genera overlapped with another healthy cohort’s core genera, with 2,678 individuals from 28 provinces of China (Lu et al., 2021). Furthermore, all core genera, except *Escherichia–Shigella*, were observed in over 90% of samples from a cohort of 483 healthy participants (Ren et al., 2023).

It has been reported that the human gut microbiota was stratified into clusters, referred to as enterotypes. Using PAM clustering based on JSD, three enterotypes were identified by the maximum CH index in our healthy samples (Figures 1C,D; Supplementary Table S11). We further identified driving genera by random forest algorithm (the area under the curve [AUC] for the receiver operating characteristic (ROC) curve: 0.98, Figures 1E,F), obtaining *Escherichia–Shigella* enterotype (E1, *n* = 250), mixture (*Bacteroides* and *Faecalibacterium*) enterotype (E2, *n* = 275), and *Prevotella* enterotype (E3, *n* = 88) (Figures 1G,H). In another Chinese cohort, 483 healthy individuals were categorized into *Bacteroides* and *Prevotella* enterotypes (Ren et al., 2023). Moreover, reports show that *Prevotella* and *Bacteroides* were two common enterotypes in Chinese populations (Lin et al., 2020). *Escherichia–Shigella* enterotype observed in our cohort is rare but consistent with the four other Chinese healthy cohorts (Liang et al., 2017; Lu et al., 2021; Lv et al., 2023; Pang et al., 2023). The alpha diversity indices (Shannon, observed species, Simpson, Pielou, ACE, and Chao1) were used to evaluate the diversity and richness of each enterotype. Notably, E2 and E3 exhibited higher Shannon, observed species, Simpson, Pielou, and ACE indices than E1 (Figures 1I–M), with no marked difference in the Chao1 index among the enterotypes (Figure 1N).

### Covariates associated with the gut microbiota composition

**Figure 2:**
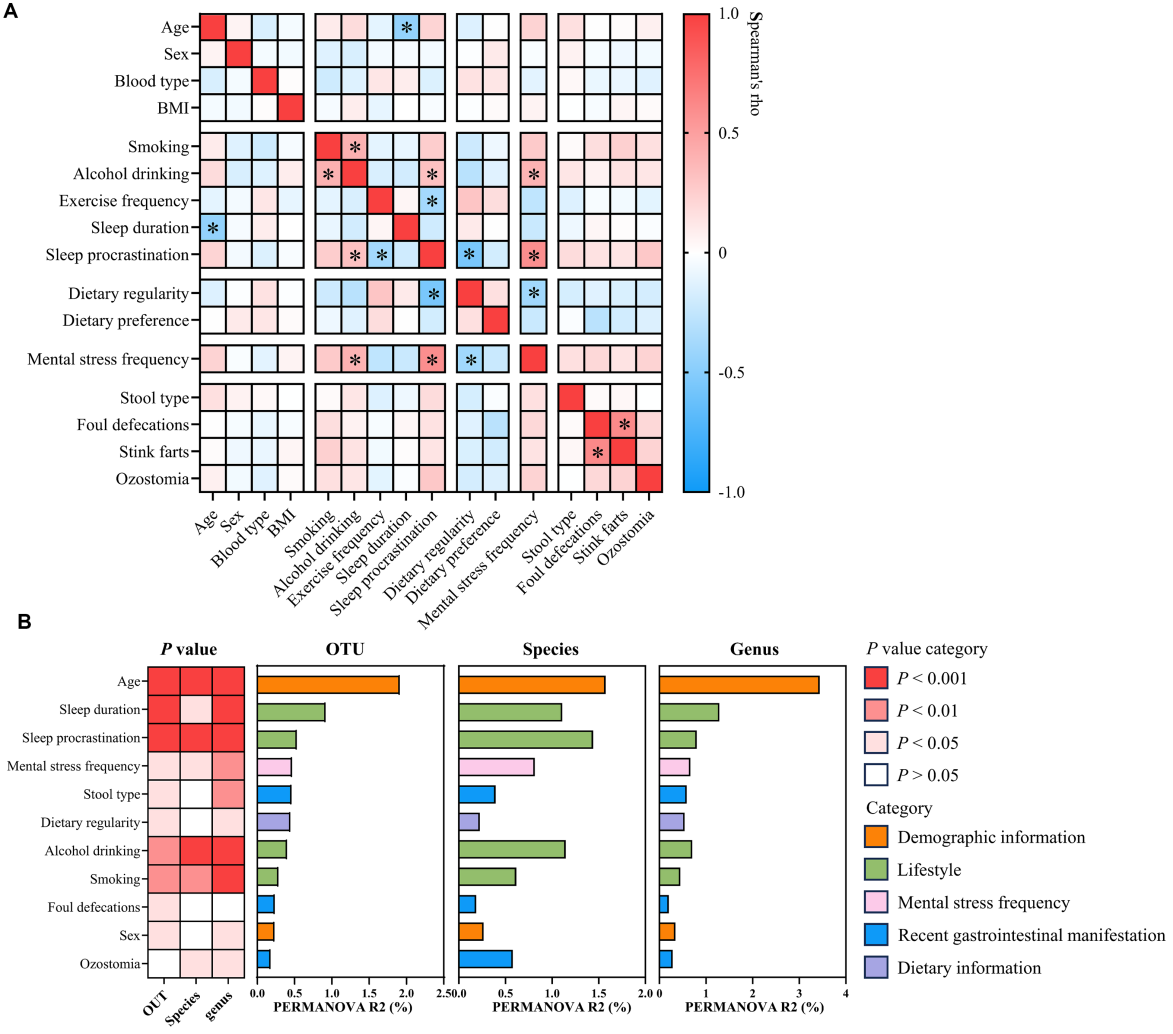
Correlation between host factors and identification of the gut microbial covariate. **(A)** Heatmap showing Spearman’s rank correlation between selected host factors. ^*^p < 0.05 and absolute Rho value ≥0.3. **(B)** Heatmap shows the *p*-value of each identified covariate as determined by PERMANOVA with Bray–Curtis dissimilarities at the OTU, species, and genus levels. Horizontal bars show inferred variance (*R*^2^) explained by each identified covariate at the OTU, species, and genus levels. Metadata categories are color-coded. If *p*-values were < 0.05, covariates were found to be statistically significant.

To investigate microbiota-associated determinants, demographic information, lifestyle, dietary information, mental stress frequency, and recent gastrointestinal manifestation of individuals were examined. Spearman’s correlation analysis revealed the potential inter-variable associations (Supplementary Tables S12, S13). Age inversely correlated with sleep duration within our cohort, suggesting a decline in sleep hours with advancing age (Figure 2A). Additionally, individuals experiencing frequent mental stress exhibited higher rates of alcohol consumption, sleep procrastination, and irregular diet (Figure 2A). Across the analysis cohort, 11 factors were identified as significant microbiota covariates at the OTU, species, and genus levels (PERMANOVA, Bray–Curtis distance, *p* < 0.05; Figure 2B; Supplementary Table S14). Age accounted for the most microbial variance, followed by several reported covariates including sex (Falony et al., 2016; Zhernakova et al., 2016; Zhang et al., 2021), mental stress frequency (Madison and Bailey, 2024), dietary regularity (Howarth et al., 2007), stool type (Falony et al., 2016; Zhernakova et al., 2016), and lifestyle factors such as sleep duration (Falony et al., 2016; Zhernakova et al., 2016), consumption of alcohol (Falony et al., 2016; Zhang et al., 2021), and smoking (Falony et al., 2016; Zhang et al., 2021). Additionally, sleep procrastination, foul defecation, and ozostomia were also associated with the gut microbiota composition (Figure 2B). It is noteworthy that age showed 8-fold more variance at the OTU level and nearly 10-fold variance at the genus level than sex (Figure 2B). Furthermore, sex has not been determined as a significant covariate at the species level in PERMANOVA analysis (Figure 2B). Overall, our results indicated that the effect size of age on the gut microbiota is far greater than that of sex.

### Association between age and the gut microbiota

The establishment of an adult-like intestinal microbial community typically occurs within the first 3–5 years of life (Odamaki et al., 2016; Vaiserman et al., 2017). Moreover, the rapid diversification of bacteria observed in infancy slows in early childhood (between 1 and 5 years of age) (Eckburg et al., 2005), and gut microbial diversity remains lower in children than in adults (Lynch and Pedersen, 2016). Furthermore, older individuals (> 60 years old) exhibited greater variability in the gut microbiome composition than younger individuals (Wilmanski et al., 2021). Therefore, our cohort was stratified into the following age groups according to previous studies (Seo et al., 2023): young children (1–5 years old, *n* = 54), children (6–17 years old, *n* = 279), young adults (18–39 years old, *n* = 82), middle-age adults (40–59 years old, *n* = 109), and elderly people (60–99 years old, *n* = 89).

**Figure 3:**
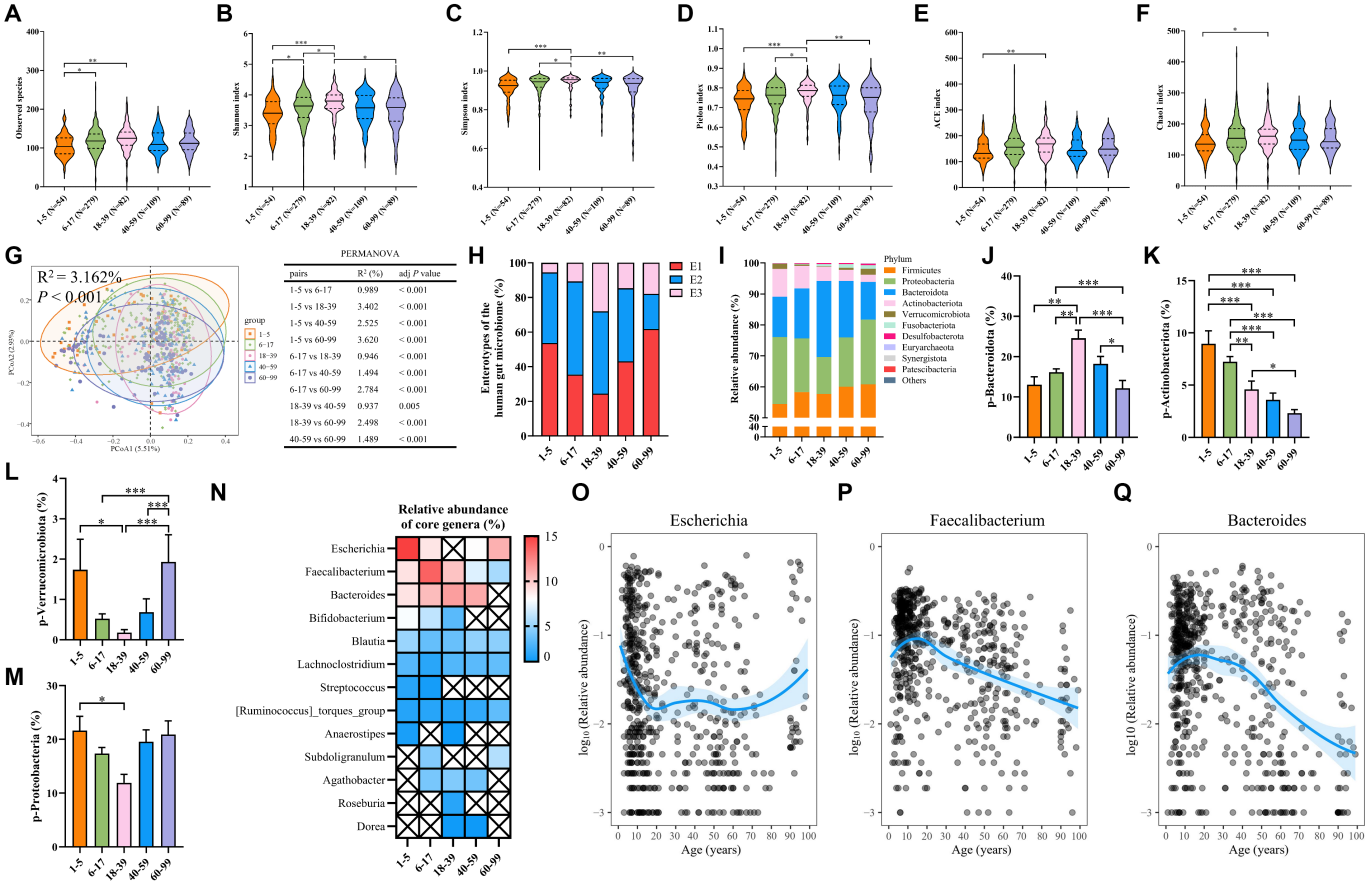
Differences in the microbiota alpha diversity, beta diversity, and composition in different age groups. **(A)** Observed species. **(B)** The Shannon index. **(C)** The Simpson index. **(D)** The Pielou index. **(E)** The ACE index. **(F)** The Chao1 index. **(G)** PCoA plot of beta diversity based on the OTU level (Bray–Curtis dissimilarities) among five age groups with a 95% ellipse. The table presents the results of PERMANOVA between groups. **(H)** The proportion of each enterotype within each age group. **(I)** The relative abundance of the top 10 abundant bacteria at the phylum level. **(J)** The relative abundance of the phylum Bacteroidota. **(K)** The relative abundance of the phylum Actinobacteriota. **(L)** The relative abundance of the phylum Verrucomicrobiota. **(M)** The relative abundance of the phylum Proteobacteria. **(N)** Heatmap showing the relative abundance of core genera in each age group. The symbol × represents bacterial genera that are not core genera of this age group. **(O–Q)** The relative abundance of the genera *Escherichia–Shigella*, *Faecalibacterium*, and *Bacteroides*. Each dot represents a sample. Fitting curves of loess regression models are indicated with blue lines. The 95% CIs are shown as blue-shaded areas. In A–F, J–M a Kruskal–Wallis test was used. Values are mean ± SEM. ^*^p < 0.05, ^**^p < 0.01, ^***^p < 0.001.

To begin with, we compared microbial community diversity and richness among five age groups by analyzing alpha diversity indices and found that the 6–17 age group has higher observed species and Shannon index than the 1–5 age group (Figures 3A,B). Moreover, all alpha diversity indices, including observed species, Shannon, Simpson, Pielou, Ace, and Chao1 indices, were higher in the 18–39 age group than in the 1–5 age group (Figures 3A–F), consistent with the previously observed lower bacterial alpha diversity in 1–5-year-old children compared to adults (Cheng et al., 2016). The Shannon and Simpson indices were used to describe the diversity of the gut microbiome, while the Pielou index was used to describe its evenness (Zheng et al., 2024). In our cohort, the gut microbiota of adults aged 18–39 years is more diverse and even compared to children aged 6–17 years, as evidenced by higher Shannon, Simpson, and Pielou indices (Figures 3B–D). In contrast, the Shannon, Simpson, and Pielou indices were lower in the 60–99 age group than in the 18–39 age group (Figures 3B–D), aligning with the observation that the microbiota in elderly people will retrogress and become less diverse (Biagi et al., 2016; Lynch and Pedersen, 2016; Ling et al., 2022). Next, we compared beta diversity using the Bray–Curtis distance at the OTU level, and PERMANOVA analysis showed a significant difference among the five age groups (Figure 3G). Intriguingly, the R^2^ values between adjacent age groups were smaller, suggesting more subtle differences in the gut microbiota composition between these groups, which further confirmed the trajectory of microbial shifts along the aging process (Figure 3G). Enterotype 1 was predominant in the 1–5 (53.7% of samples) and 60–99 (61.8%) age groups, while enterotype 2 was more common in the 6–17 (53.8%) and 18–39 (47.6%) age groups (Figure 3H).

It has been well-recognized that age is closely related to the composition of the human gut microbiota (Zhang et al., 2021; Pang et al., 2023). Therefore, we further investigated the age-related microbiota composition differences, and the relative abundance of the top 10 phyla, classes, families, genera, and species has been demonstrated (Figure 3I; Supplementary Figure S1; Supplementary Tables S5–S10). The 1–5 age group has lower phylum Bacteroidota and higher phylum Actinobacteriota than the 18–39 age group (Figure 3J), which is consistent with the reported abundance composition of the phyla Bacteroidota and Actinobacteriota in healthychildren’s intestinal tracts (Thriene and Michels, 2023). Furthermore, the phylum Actinobacteriota is more abundant in young human adults (Figure 3K) and shows a decreasing trend with age (Li et al., 2021). A similar age-dependent trend was observed in our study. Moreover, in the 40–59 and 60–99 age groups, the abundance of the phylum Bacteroidota gradually decreases, which is consistent with observations from another cross-sectional study (Pang et al., 2023), where the abundance of the phylum Bacteroidota is lower than in young adults (20–44 years), in the middle-aged (45–65 years), and elderly (66–85 years) groups. Additionally, the 18–39 age group displayed a lower abundance of the Verrucomicrobiota and Proteobacteria phyla compared to the 1–5 age group (Figures 3L,M), while the 60–99 age group had the highest abundance of the Verrucomicrobiota phylum (Figure 3L). Then, we identified the core genera in the five age groups and found that the number and abundance of core genera decrease with age (Figure 3N), which can be attributed to the increased bacterial community uniqueness (Milosevic et al., 2021; Boehme et al., 2023). The genera *Faecalibacterium*, *Blautia*, *Lachnoclostridium*, and *[Ruminococcus]_torques_group* were the shared core genera among the groups (Figure 3N). The genus *Escherichia-Shigella* was notably abundant in the 1–5 and 60–99 age groups (Figure 3O), and the genera *Faecalibacterium* and *Bacteroides* showed a decline in abundance with increasing age (Figures 3P,Q). Overall, our results indicated the existence of significant differences in alpha diversity, beta diversity, microbiota composition, and core genera abundance across different age groups.

**Figure 4:**
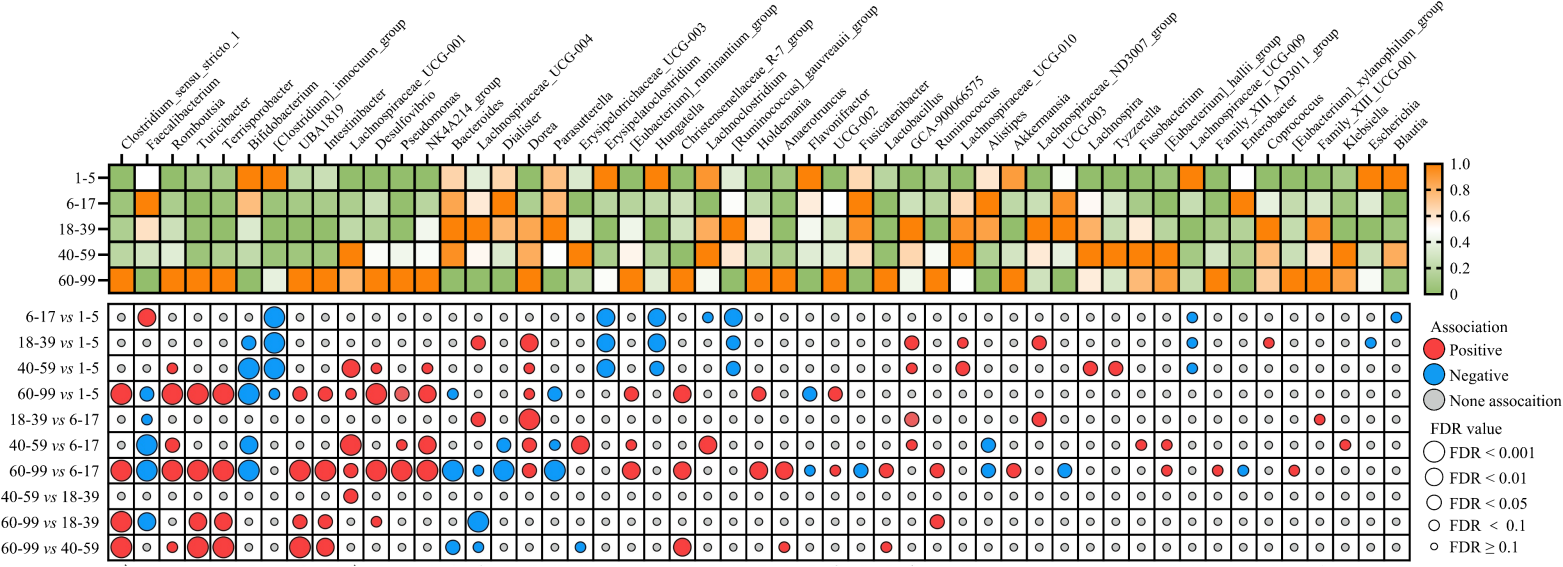
The identification of age-associated core gut microbial genera. Heatmap shows the relative abundance of age-associated genera among the five age groups. Dot plot showing the correlation and significance of age-associated genera (MaAsLin2). Colored dots indicate the directions of associations in a given model: red, significant positive associations with the age group (FDR < 0.1); blue, significant negative associations with the age group (FDR < 0.1); gray, non-significant associations (FDR ≥ 0.1). The sizes of the dots represent the FDR values from MaAsLin2 multivariate analysis (FDR ≥ 0.1, FDR  < 0.1, FDR < 0.05, FDR < 0.01, and FDR < 0.001). The greater the size, the more significant the association. MaAsLin2 models between age and genus abundance were applied by adjusting for 10 covariates (sleep duration, sleep procrastination, mental stress frequency, stool type, dietary regularity, alcohol consumption, smoking, foul defecations, sex, and ozostomia).

To further identify the age-dependent genera, MaAsLin2 multivariate analysis was used to adjust for confounding variables including sex, sleep duration, sleep procrastination, mental stress frequency, stool type, dietary regularity, alcohol drinking, smoking, foul defecations, and ozostomia (Figure 4; Supplementary Table S18). The genera *[Clostridium]_innocuum_group*, *Erysipelatoclostridium*, *Hungatella*, and *[Ruminococcus]_gauvreauii_group* were significantly more abundant in the 1–5 age group than 6–17, 18–39, and 40–99 age groups. The genera *Clostridium_sensu_stricto_1*, *Romboutsia*, *Turicibacter*, *Terrisporobacter*, *UBA1819*, *Intestinibacter*, *Lachnospiraceae_UCG001*, *Desulfovibrio*, *Pseudomonas*, *NK4A214_group*, *Dorea*, *[Eubacterium]_ruminantium_group*, *Christensenellaceae_R-7_group*, and *Holdemania*, *UCG002* were significantly more abundant in the 60–99 age group than 1–5 and 6–17 age groups. Overall, these findings highlight the significant impact of age on the composition of the gut microbiota at the genus level, with distinct microbial profiles characterizing different age brackets.

### Association between sex and the gut microbiota

**Figure 5:**
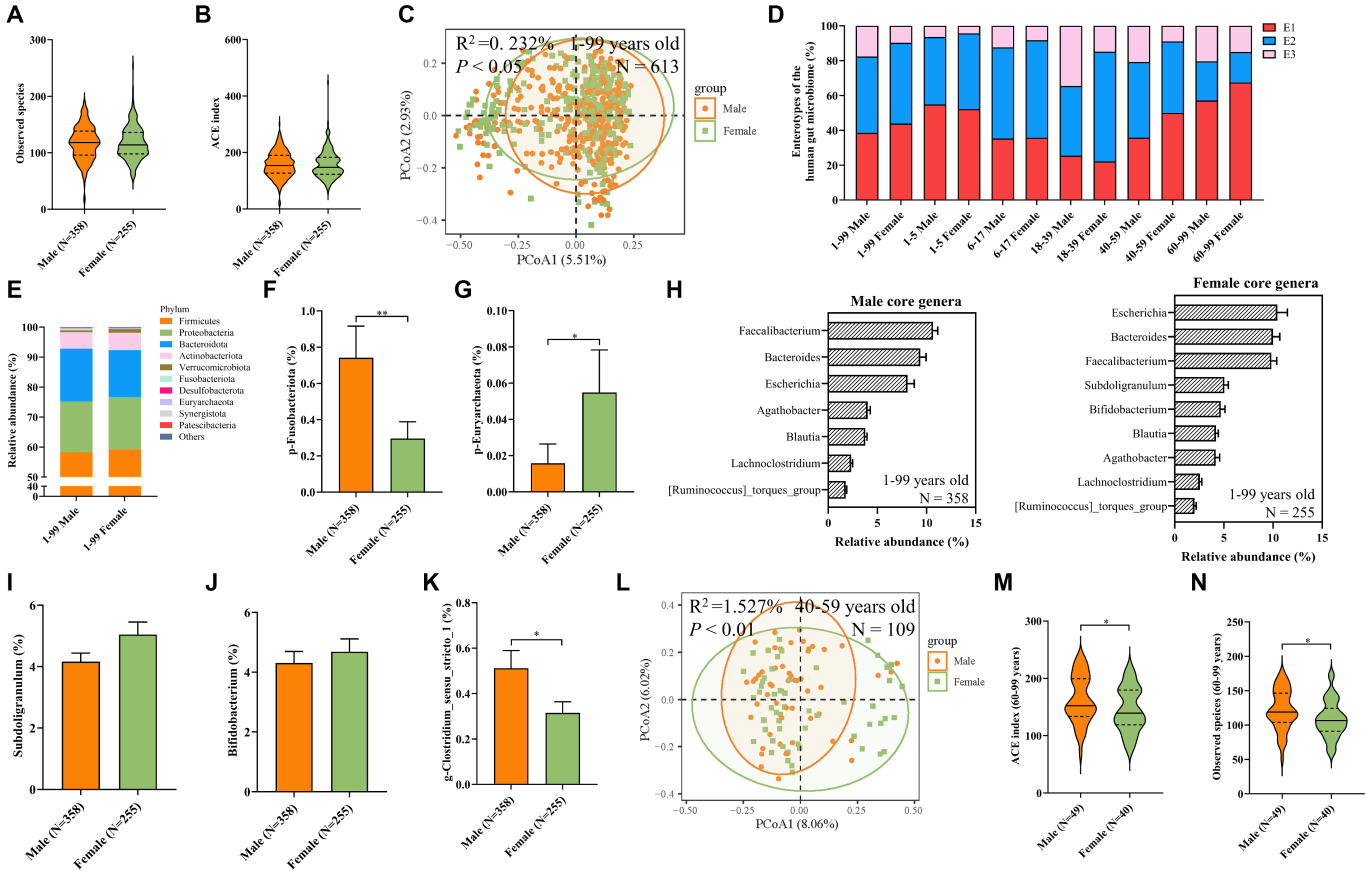
Differences of gender-associated findings in microbiota alpha diversity, beta diversity, and composition. **(A)** Observed species. **(B)** The ACE Index. **(C)** PCoA plot of beta diversity based on the OTU level (Bray–Curtis dissimilarities) among sex groups with a 95% ellipse. The upper-left corner shows the results of PERMANOVA between sex groups. **(D)** The proportion of enterotypes. **(E)** The relative abundance of the top 10 abundant bacteria at the phylum level. **(F)** The relative abundance of the phylum Fusobacteriota. **(G)** The relative abundance of the phylum Euryarchaeota. **(H)** The relative abundance of core genera of male and female groups. **(I–K)** The relative abundance of the genera *Subdoligranulum* **(I)**, *Bifidobacterium* **(J)**, and *Clostridium_sensu_stricto_1* **(K)**. In **(K)**, a MaAsLin2 model between sex and genus abundance was applied by adjusting for 10 covariates (age, sleep duration, sleep procrastination, mental stress frequency, stool type, dietary regularity, alcohol consumption, smoking, foul defecations, and ozostomia). ^*^FDR < 0.1. **(L)** PCoA plot of beta diversity based on the OTU level (Bray–Curtis dissimilarities) among sex groups at the 40–59 age layer, with a 95% ellipse. The upper-left corner shows the results of PERMANOVA between sex groups. **(M)** The ACE Index. **(N)** Observed Species. In A, B, F, G, I, J, M, and N, a two-sided Wilcoxon rank-sum test was used. Values are mean ± SEM. ^*^p < 0.05, ^**^p < 0.01.

We next investigated the role of sex in the difference of composition in the gut microbiota. To begin with, alpha diversity indices were comparable between the male and female participants (Figures 5A,B; Supplementary Figures S2B–E). However, PERMANOVA analysis based on the Bray–Curtis distance revealed significant compositional differences between the sexes (Figure 5C). The distribution of enterotypes between male and female individuals has been demonstrated (Figure 5D). Moreover, female individuals exhibited a lower abundance of the Fusobacteriota phylum and a higher abundance of the Euryarchaeota phylum compared to male individuals (Figures 5E–G). The top 10 abundant classes, orders, families, genera, and species have also been demonstrated (Supplementary Figures S3A–E). Further analysis identified two additional core genera, *Subdoligranulum* and *Bifidobacterium,* in female individuals than male individuals (Figure 5H), although their relative abundances were similar across sexes (Figures 5I,J). Utilizing MaAsLin2 multivariate analysis for sex-associated genera identification, we found associations between the genera *clostridium_sensu_stricto_1* and male individuals after adjusting for all other covariates except sex (Figure 5K; Supplementary Table S19). To mitigate the confounding effects of age, we further stratified the cohort according to predefined age groups. In each age stratum, the composition ratios of enterotypes between male individuals and female individuals are similar, except in the 18–39 age group, where female individuals have a higher proportion of E2 and a lower proportion of E3 compared to male individuals (Figure 5D). In the 1–5, 6–17, and 18–39 age groups, no significant differences in alpha diversity or beta diversity were observed between sexes (Supplementary Figures S4A–C). Similarly, alpha diversity indices were consistent between male and female participants within the 40–59 age group (Supplementary Figure S4D). However, the PERMANOVA analysis indicated a significant divergence in microbiota structure between sexes in the 40–59 age group (Figure 5L). Additionally, in the 60–99 age group, men demonstrated significantly higher ACE index and observed species than women (Figures 5M,N). Furthermore, no significant differential genera were observed between male individuals and female individuals in each age group by MaAsLin2 multivariate analysis (Supplementary Table S20). Overall, these findings suggested that sex-related differences in the gut microbiota within our cohort were subtle.

### Age-dependent dynamic alteration of the functional gut microbiota

**Figure 6:**
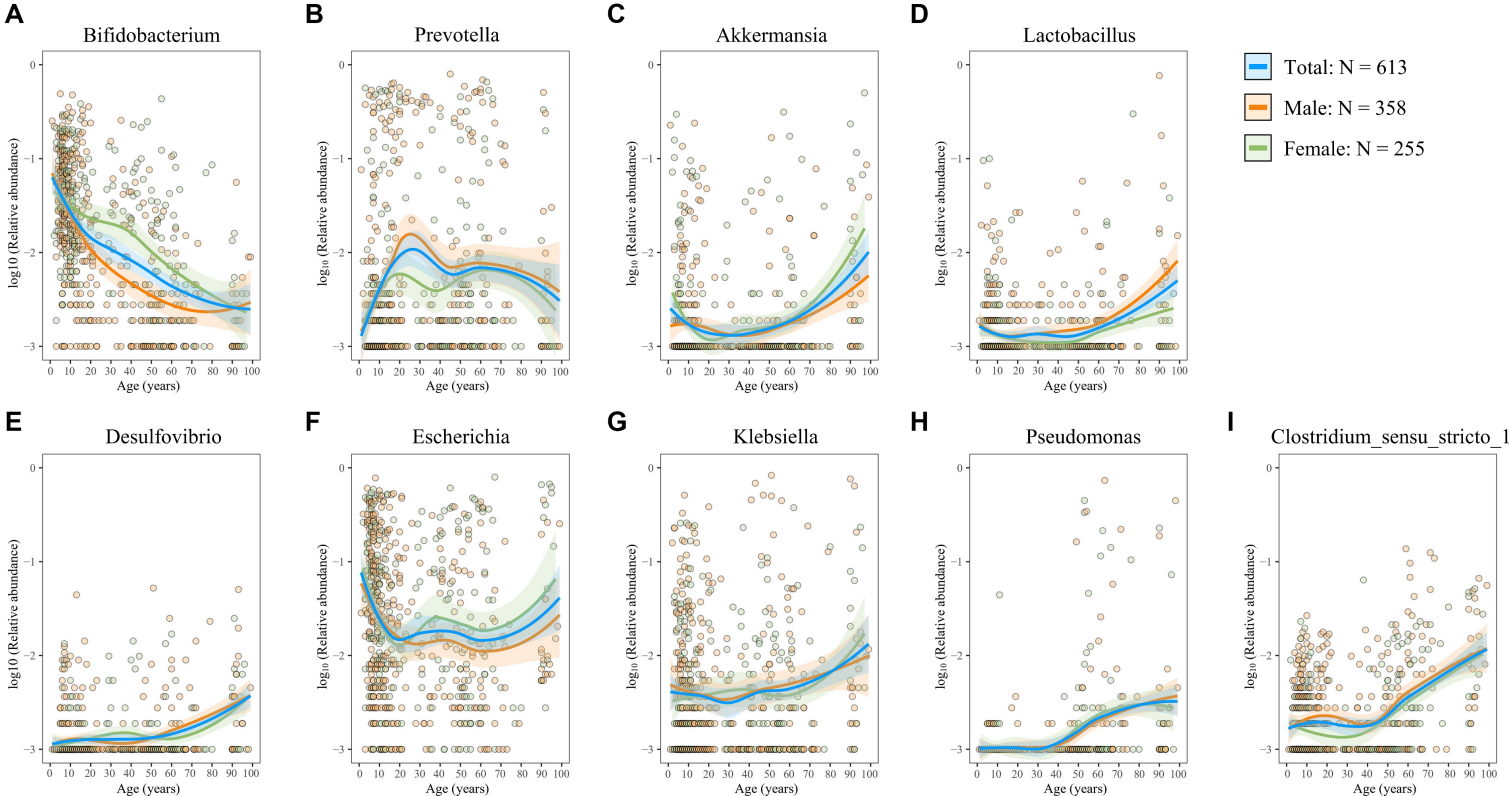
Age- and sex-related trajectories of beneficial or potentially harmful genera. **(A–I)** The relative abundance of the genera *Bifidobacterium* **(A)**, *Prevotella* **(B)**, *Akkermansia* **(C)**, *Lactobacillus* **(D)**, *Desulfovibrio* **(E)**, *Escherichia-Shigella* **(F)**, *Klebsiella* **(G)**, *Pseudomonas* **(H)**, and *Clostridium_sensu_stricto_1* **(I)**. Each dot represents a sample, with orange representing male individuals and green representing female individuals. Fitting curves of loess regression models are indicated with colored lines. 95% CIs are shown as shaded areas. Blue, 613 healthy individuals; orange, male individuals; and green, female individuals.

**Figure 7:**
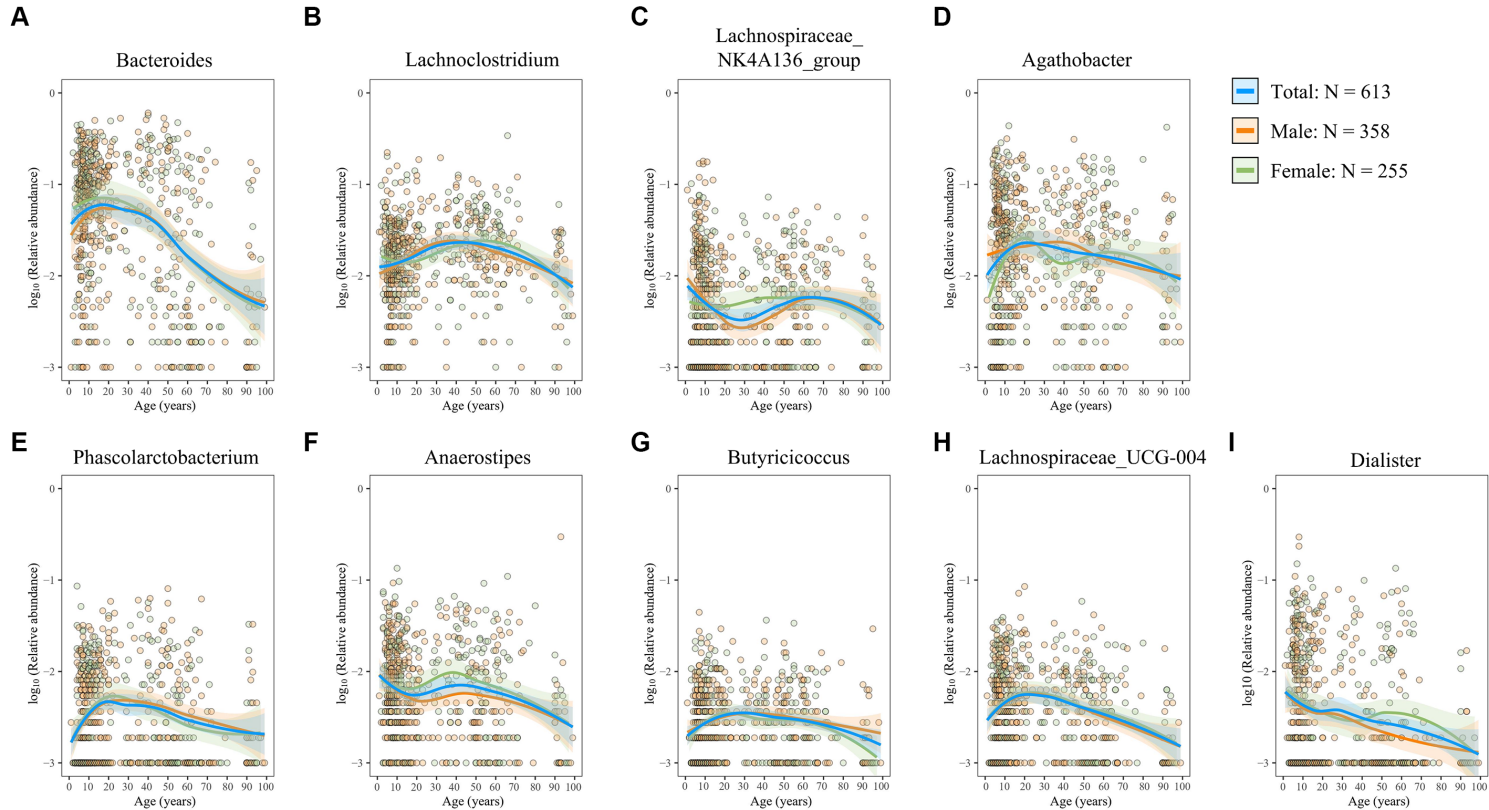
Age- and sex-related trajectories of SCFAs-producing genera. **(A–I)** The relative abundance of the genera *Bacteroides* **(A)**, *Lachnoclostridium* **(B)**, *Lachnospiraceae_NK4A136_group* **(C)**, *Agathobacter* **(D)**, *Phascolarctobacterium* **(E)**, *Anaerostipes* **(F)**, *Butyricicoccus* **(G)**, *Lachnospiraceae_UCG-004* **(H)**, and *Dialister* **(I)**. Each dot represents a sample, with orange representing male individuals and green representing female individuals. Fitting curves of loess regression models are indicated with colored lines. 95% CIs are shown as shaded areas. Blue, 613 healthy individuals; orange, male individuals; and green, female individuals.

The gut microbiota exerts its functions through interactions with the host, either directly or via its metabolites (Yao and Li, 2023). Here, we first investigated the age-related trajectories in four well-known probiotics that have beneficial effects on the host. *Bifidobacterium*, a widely distributed commensal genus within the phylum Actinobacteriota, plays a role in maintaining intestinal homeostasis and alleviating inflammation (Liu et al., 2022; Gavzy et al., 2023), thereby benefiting intestinal health. We observed a consistent decline in the genus *Bifidobacterium* with advancing age, most pronounced during infancy (Figure 6A), which is consistent with the repeatedly observed decreased trend in elderly people in several studies (Arboleya et al., 2016; Seo et al., 2023). Interestingly, this decline appeared more gradual in female individuals than in male individuals (Figure 6A). The genus *Prevotella* exhibited an initial increase followed by a subsequent decrease (Figure 6B). In contrast, the genera *Akkermansia* and *Lactobacillus* were found to be more abundant in older adults (Figures 6C,D). Furthermore, aging was associated with a marked increase in genera with potential detrimental or proinflammatory roles (Figures 6E–I), including *Desulfovibrio*, *Pseudomonas*, *Escherichia–Shigella*, *Klebsiella*, and *Clostridium_sensu_stricto_1*, which are all classic opportunistic pathogens (Rowan et al., 2010; Wyres et al., 2020; Denamur et al., 2021; Qin et al., 2022; Ye et al., 2024). Moreover, reports have shown that *Desulfovibrio*, *Pseudomonas*, *Escherichia–Shigella*, and *Klebsiella* are also LPS-producing genera (Verhaar et al., 2020; Jia et al., 2021; Mohr et al., 2022). SCFAs (e.g., butyrate, acetate, and propionate), another microbiota-derived metabolite, are critical in maintaining intestinal barrier integrity and immune balance (Martin-Gallausiaux et al., 2021). Importantly, abundant SCFAs-producing bacterial populations decreased with aging (Figures 7A–I). Moreover, the rate of decline was consistent between male and female subjects. Overall, these findings suggest a decrease in probiotic populations and an increase in potentially harmful bacteria with age, while the influence of sex on these microbial changes appears minimal.

## Discussion

In the present study, the core genera of the gut microbiota were investigated, and three enterotypes were clustered, namely, *Escherichia–Shigella*, a mixture (*Bacteroides* and *Faecalibacterium*), and *Prevotella*. Moreover, 11 covariates that explain the differences in microbiota were identified, and age exerted the strongest effect. Next, age-related differences in alpha diversity, beta diversity, and core genera were observed in our cohort. Notably, after adjusting for 10 covariates other than age, abundant genera that differed between age groups were demonstrated. Furthermore, we also demonstrated the age trajectories of several well-known beneficial genera, LPS-producing genera, and SCFAs-producing genera. Intriguingly, minimal differences in alpha diversity, beta diversity, and differentially abundant genera were observed between male individuals and female individuals.

Age is recognized as a pivotal factor influencing enterotypes in our cohort, while in the previous report, age was described as not being associated with enterotypes (Arumugam et al., 2011). However, other studies reported that age is also a factor affecting enterotypes, as indicated by the enterotypes of school-age children, adults, and elderly people (Cheng and Ning, 2019; Zhong et al., 2019; Xiao et al., 2021; Pang et al., 2023). Moreover, the enterotype describes the gut microbial community landscape, which is influenced by various factors, such as diet and BMI (Arumugam et al., 2011; Qingbo et al., 2024). Consequently, the reported enterotypes vary among cohorts from different countries and races. The *Escherichia–Shigella* enterotype observed in our cohort is rarely found in the previous reports (Lu et al., 2021) but is consistent with the four other Chinese healthy cohorts (Liang et al., 2017; Lu et al., 2021; Lv et al., 2023; Pang et al., 2023). Overall, the present study confirms the role of age in enterotypes of the gut microbiota, and the age-related enterotype alterations require further study.

Associations between age and gut microbiota diversity and composition have been observed both in our study and in others (de la Cuesta-Zuluaga et al., 2019; Zhang et al., 2021; Pang et al., 2023). However, most studies’ understanding of age-mediated gut microbiota differences stems from comparisons within isolated age segments of adults or pediatric individuals, while the present study encompasses a healthy demographic population ranging from 1 to 99 years old, which is beneficial for a comprehensive understanding of the age trajectory of the gut microbiota. Moreover, beta diversity analysis confirms the age-related microbial structure differences. Furthermore, higher alpha diversity indices were observed in younger adults than those in children and elderly people in our study. Importantly, after adjusting for 10 covariates other than age, we identified numerous differentially abundant bacterial genera between age groups. Indeed, due to the close association between age and gut microbiota composition, researchers have attempted to predict age through the microbiota difference across age groups in healthy individuals (Seo et al., 2023). However, it should be noted that the changes observed in the gut microbiota associated with age are not entirely consistent due to biological differences such as race, country, culture, and lifestyle (Seo et al., 2023). Therefore, although the gut microbiota can relatively well predict the age of healthy individuals, its applicability across different populations still requires further investigation.

Abundant potential SCFAs-producing genera were observed to decrease with aging, which is consistent with the previous observation that SCFAs and SCFAs-producing microbiota decreased with aging (Woodmansey et al., 2004; Salazar et al., 2013, 2019). Therefore, most studies suggest that supplementation of SCFAs, which can contribute to intestinal homeostasis and health (Zhang et al., 2022), in healthy elderly individuals is also essential. Additionally, SCFAs are beneficial for the integrity of the gut barrier, which tends to weaken with aging (Wu et al., 2023). Therefore, the supplementation of SCFAs should be given greater consideration in elderly people than in the young, and elderly people should consume fiber-rich diets (Li et al., 2024), incorporate prebiotics such as fructooligosaccharides that promote SCFAs production (Kumar et al., 2012), intake SCFAs-producing probiotics, or directly supplement with SCFAs to facilitate health.

The age trajectories of four well-recognized probiotics were also investigated in the present study. The abundance of Bifidobacterium decreases along the age trajectory from infancy, whereas *Akkermansia* and *Lactobacillus* demonstrate an increasing trend with advancing aging. Moreover, the genus *Prevotella* is enriched in young adults and decreases with aging. First, it should be noted that intestinal health is the outcome of the collective action of various probiotics. Our speculation is that the types of probiotics that maintain health vary across different age stages, and their inter-crosstalk sustains human health collectively. Second, other factors, such as diet, may also induce probiotic alteration, although dietary preference for meat or vegetables did not account for microbial variation in our study. Previous studies have shown that the probiotic *Prevotella* is an indicator of favorable postprandial glucose metabolism (Asnicar et al., 2021), and the *Prevotella* enterotype is related to carbohydrate dietary patterns (Wu et al., 2011). Third, although there is a significant correlation between age and the abundance of probiotics, the causal relationship between aging and probiotics remains unclear. For example, the decline of *Bifidobacterium* with aging was associated with a reduction in adhesion to the intestinal mucosa, but it is not clear whether it is due to alterations in the microbiota or intestinal microenvironment of elderly people (Arboleya et al., 2016). Additionally, reports show that supplementing reduced *Bifidobacteria* can extend lifespan (Shujie et al., 2021), and *Akkermansia* and *Lactobacillus* are two hallmarks of healthy aging [56, 57], suggesting that regulating probiotics may contribute to the youthful state of the intestine. However, the causal relationship between aging and probiotic abundance remains elusive, necessitating further research.

A slight increase in the abundance of the genera *Pseudomonas*, *Escherichia–Shigella*, *Klebsiella*, and *Desulfovibrio* with aging was observed in our study, which can produce LPS (Verhaar et al., 2020; Jia et al., 2021; Mohr et al., 2022). Previous studies reported that gut microbiota LPS can accelerate inflammaging in mice (Kim et al., 2016). Considering that the elderly individuals included in our study were rigorously selected as healthy subjects, the slight increase in the LPS-producing genus does not appear to affect their normal physiological functions. The underlying reasons and implications warrant further investigation. Previous studies report that small amounts of LPS can induce endotoxin tolerance (Yuan et al., 2016), which may explain the protective effects of increased LPS-producing bacteria in elderly people. Moreover, alterations in the gut environment that align with aging, such as pH shifts and alterations in gut motility, can favor the growth of different bacterial populations (Kedia et al., 2018), including those that produce LPS. Taken together, the cause and significance of the slight increase in LPS-producing bacteria with aging merit further exploration.

In our cohort, PERMANOVA analysis showed that age explains 8-fold more variance at the OTU level and nearly 10-fold variance at the genus level than sex, while sex has not been determined as a significant covariate at the species level. Moreover, when gender was considered a grouping factor, no obvious differences between male individuals and female individuals were observed in the age-related changes of alpha diversity. Numerous studies have shown that enterotype is less affected by gender (Kim et al., 2020; Matusheski et al., 2021). Consistently, there is a similar distribution of enterotypes across sexes overall in our observation. Even after adjusting for covariates other than sex, only the genus Clostridium_sensu_stricto_1 was observed to increase in male individuals significantly. Notably, after further stratification according to age group, there are minimal differences between sexes in alpha diversity, beta diversity, and microbial composition. In conclusion, the effect size of age on the gut microbiota is much greater than that of sex in our cohort.

There are several limitations to our study. First, as a retrospective study design, comprehensive physiological indices and detailed dietary information, which are essential in understanding factors responsible for or affected by the singular gut microbiota characteristics uncovered here, are absent in the present study. Moreover, metabolites originating from the microbiota are significant mediators of its beneficial or detrimental health effects. Although we observed numerous alterations in SCFAs-producing bacteria, the quantities of SCFAs were not further directly quantified in this study. In addition, the region-related microbiota differences also need to be considered, while our sample size to further group our Chinese individuals was insufficient. For instance, samples from Liaoning (*n* = 1), Inner Mongolia Autonomous Region (*n* = 1), and Yunnan (*n* = 1) provinces are insufficient and lacking representation. Therefore, to investigate region-mediated microbial differences, we further grouped the samples: Hubei province (*n* = 494) and non-Hubei province (*n* = 119). The PCoA result indicates that the gut microbiota structures of the two groups are similar (Supplementary Figure S5). Importantly, after adjusting for 11 covariates, such as age and gender, the PERMANOVA analysis revealed that the region did not mediate significant differences at the OTU, genus, and species levels between the two groups in the present study (Supplementary Figure S5). More samples from various provinces of China need to be further collected in the future to investigate the inter-provincial microbiota differences. Alternatively, a comparative analysis of Chinese samples with those from other countries at the national level would help to clarify region-related microbiota differences. Furthermore, it is worth noting that our study employed 16S rRNA sequencing as the methodological approach. However, the sequencing depth does not allow precise identification at the species level. Even within the same genus, significant metabolic and functional differences exist among different species. Therefore, future research should utilize metagenomic assays to elucidate microbial functional differences related to age and gender at the species level.

## Conclusion

This study reports features of the gut microbiome associated with age and gender in a healthy Chinese population. Age exerts stronger impacts on microbial alpha diversity, beta diversity, and composition than sex. Moreover, we demonstrated the dynamic alterations of gut probiotics or LPS-producing microbiota across the aging process, suggesting the need to supplement beneficial bacteria, particularly those producing SCFAs, according to age. In conclusion, our results elucidate the age trajectory of the gut microbiota, contributing to the understanding of the microbiota spectrum thereby implementing precise microbiota intervention strategies.

## Data availability statement

The 16S rRNA sequencing data in this study are available in the Sequence Read Archive (SRA) under accession number PRJNA1114722.

## Ethics statement

The studies involving humans were approved by Ethics Committee of Union Hospital, Tongji Medical College, Huazhong University of Science and Technology (No. 2023-179). The studies were conducted in accordance with the local legislation and institutional requirements. Written informed consent for participation in this study was provided by the participants’ legal guardians/next of kin.

## Author contributions

JW: Investigation, Methodology, Project administration, Software, Visualization, Writing – original draft, Writing – review & editing. HS: Methodology, Project administration, Resources, Supervision, Validation, Writing – original draft, Writing – review & editing. YL: Investigation, Methodology, Supervision, Writing – review & editing. JH: Investigation, Validation, Writing – review & editing. XX: Investigation, Writing – review & editing. ZX: Investigation, Writing – review & editing. PY: Methodology, Writing – review & editing. WQ: Data curation, Writing – review & editing. TB: Conceptualization, Funding acquisition, Writing – original draft, Writing – review & editing. XH: Conceptualization, Data curation, Writing – original draft, Writing – review & editing.

## Footnotes

^1^http://enterotype.embl.de/enterotypes.html

^2^https://github.com/biobakery/biobakery/wiki/maaslin2
