# Supplementary material for: Age over sex: evaluating gut microbiota differences in healthy Chinese populations
Source: Front Microbiol. 2024 Jun 21;15:1412991. doi: 10.3389/fmicb.2024.1412991 (PMC11224521; doi:10.3389/fmicb.2024.1412991)
Supplement: Supplementary file 2 [file Table_2.DOCX]

**Age over sex: evaluating gut microbiota differences in healthy Chinese populations**

Jiacheng Wu^1^^†^, Hexiao Shen^2†^, Yongling Lv^2^, Jing He^1^, Xiaotian Xie^1^, Zhiyue Xu^1^, Pengcheng Yang^1^, Wei Qian^1^, Tao Bai^1,*^, Xiaohua Hou^1*^

**^†^These authors have contributed equally to this work**

**Affiliations**

^1^Division of Gastroenterology, Union Hospital, Tongji Medical College, Huazhong University of Science and Technology, Wuhan, Hubei 430062, China;

^2^School of Life Science, Hubei University, Wuhan, Hubei 430062, China.

***Correspondence**

Professor Xiaohua Hou, MD, PhD, Division of Gastroenterology, Union Hospital, Tongji Medical College, Huazhong University of Science and Technology, 1277 Jiefang Avenue, Wuhan, Hubei 430022, China. Email: houxh@hust.edu.cn

Professor Tao Bai, MD, PhD, Division of Gastroenterology, Union Hospital, Tongji Medical College, Huazhong University of Science and Technology, 1277 Jiefang Avenue, Wuhan, Hubei 430022, China. Email: [drbaitao@126.com](mailto:drbaitao@126.com)





Figure S1 The composition of gut microbiota in different age groups. Relative abundance of top 10 abundant bacteria at the class (A), order (B), family (C), genus (D), and species (E) level.





Figure S2 The alpha diversity between male and female individuals. (A) The percentage of male and female individuals in each age layer. (B) Simpson index. (C) Chao1 index. (D) Pielou index. e Shannon index.





Figure S3 The composition of gut microbiota in different sex groups. Relative abundance of top 10 abundant bacteria at the class (A), order (B), family (C), genus (D), and species (E) level.





Figure S4 The alpha diversity and beta diversity between male and female individuals in each age layer. (A) The alpha diversity indices and PCoA plot of beta diversity between males and females aged 1-5 years. (B) The alpha diversity indices and PCoA plot of beta diversity between males and females aged 6-17 years. (C) The alpha diversity indices and PCoA plot of beta diversity between males and females aged 18-39 years. (D) The alpha diversity indices between males and females aged 40-59 years. (E) The alpha diversity indices and PCoA plot of beta diversity between males and females aged 60-99 years.





Figure S5 The beta diversity between Hubei and non-Hubei group individuals. (A) The PCoA plot and PERMANOVA analysis.
